# Overexpression of lactate dehydrogenase-A in human intrahepatic cholangiocarcinoma: its implication for treatment

**DOI:** 10.1186/1477-7819-12-78

**Published:** 2014-03-31

**Authors:** Yaping Yu, Minqi Liao, Ruiwen Liu, Jian Chen, Hao Feng, Zan Fu

**Affiliations:** 1 Department of Hepatobiliary Surgery, Kunshan People’s Hospital Affiliated to Jiangsu University, Kunshan 215300, Jiangsu, China; 2 Division of Minimally Invasive Surgery, Department of General Surgery, First Affiliated Hospital of Nanjing Medical University, NO. 300, Guangzhou Road, Nanjing 210029, China

**Keywords:** Intrahepatic cholangiocarcinoma, Lactate dehydrogenase-A, RNA interference

## Abstract

**Background:**

Previous studies have shown that lactate dehydrogenase-A (LDH-A) is strongly expressed in several malignancies, that LDH-A expression is associated with poor prognosis, and that LDH-A inhibition severely diminishes tumorigenicity. However, little is known about the implications of LDH-A expression in intrahepatic cholangiocarcinoma. The purpose of this study was to investigate the expression of LDH-A and to clarify its effect on intrahepatic cholangiocarcinoma.

**Methods:**

We studied the expression of LDH-A in tissue samples from patients with intrahepatic cholangiocarcinoma (n = 54) using the ultrasensitive surfactant protein (S-P) immunohistochemical method. We then inhibited LDH-A using small hairpin RNA (shRNA) in the cholangiocarcinoma cell line HuCCT-1 *in vitro* to study the role it plays in promoting growth and escaping apoptosis.

**Results:**

We report that LDH-A was overexpressed in 52 of 54 (96%) paraffin-embedded cancer tissue samples and 0 of 54 para-carcinoma tissue samples. Reduction of LDH-A by RNA interference (RNAi) inhibited cell growth and induced apoptosis in HuCCT-1 cells. This result correlated with the elevation of cytoplasmic reactive oxygen species (ROS) levels.

**Conclusions:**

LDH-A expression is closely correlated with histopathological variables of intrahepatic cholangiocarcinoma, indicating that LDH-A may serve as a new treatment target.

## Background

Intrahepatic cholangiocarcinoma (ICC) is a devastating malignancy that arises from the epithelial lining of the intrahepatic bile ducts; recent data have demonstrated an increase in the incidence and mortality of ICC worldwide [1]. Due to the difficulty in detecting ICC at the early stage and the disappointing chemotherapy strategies offered currently, the prognoses of patients with advanced cholangiocarcinoma are very poor [2,3]. Therefore, new insights into cancer cell-specific biological pathways are urgently needed to promote development of rationally targeted and more effective therapeutics.

Even under aerobic conditions, cancer cells preferentially undergo glycolysis followed by fermentation; this phenomenon is known as aerobic glycolysis (or the Warburg effect) [4]. Lactic dehydrogenase A, LDH-5 (LDH-A), the NADH-dependent enzyme that catalyzes the conversion of pyruvate to lactate, is a key glycolytic enzyme for aerobic glycolysis. Inhibition of LDH-A has been shown to cause a blockade of aerobic glycolysis of tumor cells [5] and to severely diminish the tumorigenicity of neu-initiated mammary tumor cells [6,7]. Previous studies have shown that LDH-A is strongly expressed in gastric, pulmonary, gynecologic, and breast malignancies associated with poor prognoses [8-10]. However, the expression of LDH-A in clinical tissue samples of ICC has not been previously described, and the effects of LDH-A in ICC have been unclear.

In metazoan cells, reactive oxygen species (ROS) are generated by mitochondria during oxidative phosphorylation (OXPHOS) [11]. Under normal conditions, ROS appear to promote cell growth. In contrast, they activate and modulate apoptosis when cells are under stress [12]. As a key effector of cell death, ROS inflict oxidative damage on cellular proteins, lipids, and nucleic acids. In advanced tumors, however, these agents are downregulated, and consequent cell-death signaling is suppressed [13]. Considering that aerobic glycolysis diminishes cellular ROS in yeast [14], we hypothesized that silencing LDH-A by blocking aerobic glycolysis might upregulate ROS levels and induce apoptosis of ICC.

In the ICC tissues examined in the present study, LDH-A was overexpressed and the expression of LDH-A was associated with tumor differentiation. Reduction of LDH-A by RNA interference (RNAi) inhibited cell growth in liquid culture and induced cell apoptosis. Furthermore, the inhibition of LDH-A elevated cytoplasmic ROS levels. In other words, overexpression of LDH-A in cholangiocarcinoma might be associated with ROS downregulation. Taken together, these results suggest that LDH-A is a potential therapeutic target for ICC treatments.

## Methods

### Reagents and cell lines

Cholangiocarcinoma cell lines (HuCCT-1 cells) were purchased from the Chinese Academy of Sciences. Dulbecco’s modified Eagle’s medium (DMEM), phosphate-buffered saline (PBS), fetal bovine serum (FBS), non-essential amino acid solution (NEAA 10 mM, 100×), penicillin-streptomycin solution (10,000 units/ml penicillin and 10,000 μg/ml of streptomycin) and trypsin-ethylenediaminetetraacetic acid (EDTA) solution (0.05% trypsin, 0.53 mM EDTA) were obtained from Invitrogen-Life Technologies (Carlsbad, CA, USA).

### ICC tissue samples

Paraffin-embedded samples of ICC were obtained from 42 patients who underwent surgical excision without any prior therapy between 2010 and 2011 at The First Affiliated Hospital of Nanjing Medical University. The para-carcinoma tissue we choose were 1 cm or more away from the cancerous tissue. Histological diagnoses and grading were performed on hematoxylin-eosin-stained tissue sections. All patients had given informed consent. The study was approved by the Institutional Review Board of The First Affiliated Hospital of Nanjing Medical University. The patients’ clinical data are listed in Table 1. A total of 32/42 patients were mass-forming, 8/42 were periductal-infiltrating, and 2/42 were intraductal-growing cholangiocarcinomas. Histologically, all patients enrolled had tubular adenocarcinomas.

**Table 1:** Correlation between LDH-A expression level and clinicopathologic characteristics in ICC patients

**Variables**			**LDH-A**	
		**High**	**Low**	***P *** **value**
Sex	Male	10	10	0.554
	Female	13	9	
Age, years	>55	9	13	0.058
	≤55	14	6	
Tumor size, cm	>6	9	9	0.591
	≤6	14	10	
Grade	1	6	11	0.037
	2/3	17	8	
Lymph node	Yes	9	4	0.207
metastasis	No	14	15	

*ICC*, intrahepatic cholangiocarcinoma; *LDH-A*, lactate dehydrogenase-A.

### Immunohistochemistry

First, the 4-μm tissue sections were deparaffinized with xylene and rehydrated. Antigen retrieval was performed by autoclaving for 10 minutes. Endogenous peroxidase activity was quenched with methanol and hydrogen peroxide (2.5% to 3.5%) for five to eight minutes, then the sections were washed for three minutes in PBS three times. The primary antibody against LDH-A (dilution 1:100; ab9002, Abcam, Cambridge, UK) was applied at room temperature and the sections placed in the refrigerator at 4°C overnight.

Following washing with PBS (three times for three minutes), tissue sections were incubated with a rabbit anti-sheep IgG (Bioworld Products, Visalia, CA, USA) at 37°C for 30 minutes. Then the sections were washed in PBS. Streptavidin peroxidase reagent was applied for 10 minutes. The color was developed via a three- to five-minute incubation with 3,3′-diaminobenzidine (DAB) solution. Finally, the sections were counterstained with hematoxylin for five to eight minutes and mounted on glass specimen plates.

Treated tissue sections were observed under an optical field with 200× magnification. According to the percentage of positive cells in the cytoplasm and the nuclear expression, which was assessed in a grading system [15], two LDH-A expression levels were established: strong cytoplasmic expression in >50% of cancer cells or nuclear expression in >10% of cancer cells was defined as high expression; otherwise, nuclear expression was considered low.

### Silencing LDH-A with RNA interference

The target sequence for LDH-A small interfering RNA was 5′-GGAGAAAGCCGUCUUAAUU-3′. The control nucleotide sequence of small interfering RNA was 5′-GTTCTCCGAACGTGTCACGT-3′. This random sequence is not related to LDH-A mRNA. The pGPU6/Neo eukaryotic expression vector with LDH-A siRNA (shRNA) or LDH-A siRNA con (NC) was transiently transfected into the HuCCT-1 cell line using Lipofectamine2000 reagent according to the manufacturer’s protocol (Invitrogen). The interference effect was verified by RT-PCR and western blot assay at 48 and 72 hours post-transfection.

### Real-time PCR

The mRNA levels of LDH-A and β-actin were analyzed using real-time PCR. Briefly, 2 μg of high-quality RNA was processed directly to cDNA in a total volume of 25 μl using a reverse transcription kit (Takara, Dalian, China) according to the manufacturer’s instructions. Amplification reactions were performed in a 50-μl volume of SYBR Premix Ex Taq (Takara, DRR041A). The PCR reaction included the following components: 10 pmol of primer, 2 mM MgCl_2_, 200 μM dNTP mixture, 0.5 U of Taq DNA polymerase and universal buffer. The thermal cycling conditions were as follows: 95°C for 30 seconds; 40 cycles at 95°C for 5 seconds, 60°C for 31 seconds; and a final dissociation stage. The specificity of amplification was examined by melting curve analysis and by electrophoresis in 2% agarose gel. All of the reactions were performed in triplicate in an ABI PRISM 7300 real-time PCR system. The primer sequences for the human LDH-A gene were as follows: forward primer, 5′-AGCCCGATTCCGTTACCT-3′ and reverse primer: 5′-CACCAGCAACATTCATTCCA-3′. As an internal standard, a fragment of human β-actin was amplified by PCR using the following primers: forward primer, 5′-CCAAAGCAACCGTGAGGA-3′ and reverse primer, 5′-CCAGAGGCGTACAGGACA-3′. Data are presented as the fold change of LDH-A expression in each tumor tissue relative to its paired control group tissue after normalization with β-actin.

### Western blot analysis

Cells were harvested and lysed in a buffer containing 50 mM Tris–HCl pH 7.5, 150 mM NaCl, 2 mM EDTA, 1% Triton and 1 mM PMSF for 20 minutes on ice. Lysates were cleared by centrifugation at 17,970 × *g*, 4°C for 10 minutes. Supernatants were collected, and protein concentrations were determined using a Bradford assay (Beyotime, Wuhan, China). The proteins were then separated with an SDS/polyacrylamide gel and transferred to a polyvinylidene fluoride (PVDF) membrane (Bio-Rad, Berkeley, CA, USA). After blocking in PBS with 5% non-fat dry milk for 1 hour, the membranes were incubated overnight at 4°C with the primary antibodies in PBS with 5% non-fat dry milk. Anti-LDH-A rabbit antibody (1:1000, Cell Signaling Technology, Danvers, MA, USA) and anti-β-actin polyclonal antibody (1:2000, Bioss International, Shanghai, China) were used. Membranes were extensively washed with PBST and incubated with horseradish peroxidase-conjugated secondary anti-mouse antibody or anti-rabbit antibody (1:2,000, Bio-Rad). After additional washes with PBST, antigen-antibody complexes were visualized with a chemiluminescence kit (Pierce, Thermo-Fisher Scientific, Waltham, MA, USA).

### Cell viability assay

To analyze the effect of LDH-A on the viability of HuCCT-1, a total of 3 to 5 × 10^3^ cells per well were seeded in 96-well plates. After 12 hours, the cells were transfected with shRNA or NC plasmids and incubated for 96 hours. Cell viability was determined using a Cell Counting Kit-8 assay according to the manufacturer’s protocol (Dojindo Laboratories, Kumamoto, Japan) at 24, 48, 72, and 96 hours after transfection.

### Cell apoptosis assay

To analyze apoptosis, cells with or without treatment were stained with annexin-V–fluorescein isothiocyanate (FITC) and 7-aminoactinomycin D (7-AAD), according to the manufacturer’s instructions (Becton Dickinson Biosciences, San Jose, CA, USA). Data acquisition and analyses were performed using a FACScan flow cytometer with Cell-Quest software (BD Biosciences). Cells that were positively stained by annexin-V-FITC only (early apoptosis) and were positive for both annexin-V–FITC and 7-AAD (late apoptosis) were quantitated, and both subpopulations were considered as dead cells.

### Measurement of levels of intracellular ROS

Intracellular ROS production was measured by staining cells with carboxy-H2 dichlorofluorescin diacetate (DCFDA; molecular probes) according to the manufacturer’s instructions (Beyotime, Wuhan. China). Briefly, treated HuCCT-1 cells were cultured in the presence of 20 μM carboxy-H2DCFDA for 30 minutes. After removing the culture medium, cells were washed twice with PBS, resuspended in growth medium and measured by flow cytometry.

### Statistics

Results are expressed as mean ± standard deviation (SD). The *χ*^2^ test was used to test relationships between categorical variables including age, gender, tumor size, grade, lymph node metastasis and the expression of LDH-A. Independent-sample t-tests were used to compare LDH-A mRNA, the proliferation, apoptosis, and ROS levels between the RNAi and the negative control (NC) groups. Statistical significance was considered *P* <0.05.

## Results

### Immunohistochemical expression of LDH-A and its association with histopathological variables

**Figure 1:**
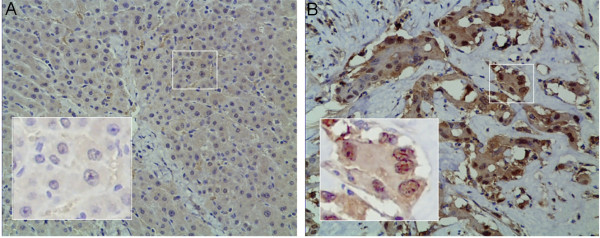
**Immunohistochemical staining of lactate dehydrogenase-5 (LDH-A) in (A) para-carcinoma tissue and (B) tissue sections from human ICC.** (B) Corresponding (original magnification, 200×). (A) Weak cytoplasmic and nuclear expression. (B) Strong cytoplasmic and nuclear expression. ICC, intrahepatic cholangiocarcinoma.

First, we studied the expression of LDH-A in ICC tissue samples using the S-P immunohistochemical method. Immunohistochemical expression was observed to be nuclear and/or cytoplasmic (Figure 1). Of 42 ICC tissue samples, 23 (54.8%) demonstrated high LDH-A reactivity as categorized using the grading system previously mentioned [15]. In 16 ICC tissue samples, adjacent non-cancerous tissues were consistently unreactive to LDH-A. The association of LDH-A with histopathological variables is shown in Table 1. LDH-A expression was positively correlated with histopathological variables (*P* <0.05). That is, the overexpression of LDH-A was more prevalent in poorly differentiated ICC cells. However, no significant association was noted between LDH-A expression and lymph-node metastasis.

### Reduction of LDH-A inhibited growth and induced apoptosis of HuCCT-1

**Figure 2:**
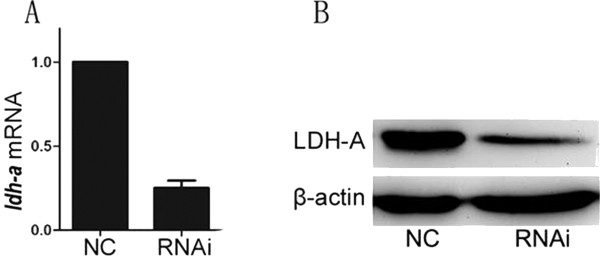
**Knock-down of LDH-A inhibits growth and induces apoptosis in the HuCCT-1 cell line. (A)** LDH-A expression was analyzed after transfection by RT-PCR, with actin as loading control. The figure shows that RNAi induced a specific decrease in LDH-A expression after transfection. **(B)** Western blot assay also shows a specific decrease in LDH-A expression after transfection, with actin as loading control. LDH-A, lactate dehydrogenase A; RNAi, RNA interference.

**Figure 3:**
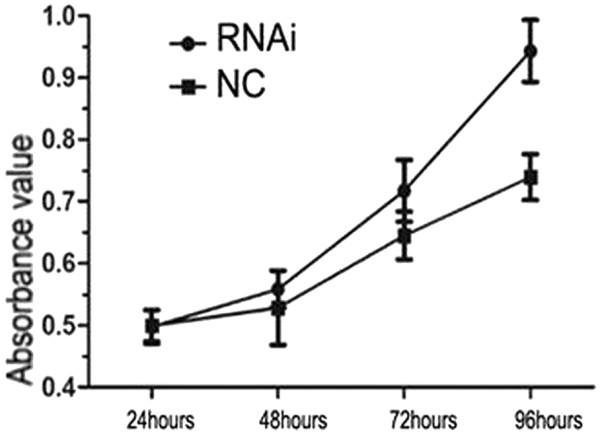
**LDH-A knockdown inhibits HuCCT-1 cell growth.** Relative cell numbers at 72 and 96 hours post-transfection for RNAi versus NC have a *P* value of 0.008 and 0.004, respectively. LDH-A, lactate dehydrogenase A; NC, negative control; RNAi, RNA interference.

**Figure 4:**
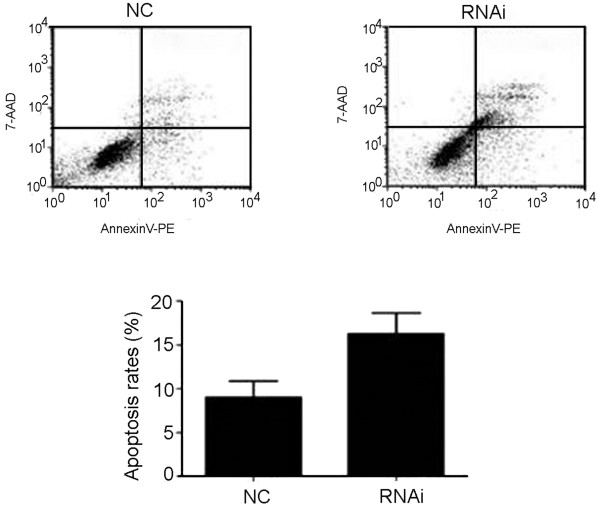
**Cell death was determined by flow cytometry of annexin V- and 7-AAD-stained cells at 72 hours post-transfection with shLDH-A (RNAi) or siControl (NC).** The histogram (right) represents the average percentage (±SD) of dead cells. The number of dead cells treated with shLDH-A (RNAi) compared with the control group (NC) has a *P* value of <0.05 using Student’s t test. 7-ADD, 7-aminoactinomycin D; RNAi, RNA interference; SD, standard deviation; sh, small hairpin.

To investigate the effect of aberrant LDH-A expression in cholangiocarcinoma, RNAi experiments were performed using the HuCCT-1 cell line. We examined the expression of LDH-A at its mRNA and protein levels by performing RT-PCR and western blotting analyses in the HuCCT-1 cell line (Figure 2A, B). The CCK-8 assay showed that the proliferation rates of the HuCCT-1 LDH-A knocked-down cell line (RNAi group) were slower than those of the negative control HuCCT-1 cell lines (NC group) (Figure 3). The absorbance value of transfected cells at 72 and 96 hours decreased markedly compared to the negative control cells (*P* <0.01). The apoptosis rates of the RNAi group (25.89% ± 1.86%) obviously increased compared to those of the NC group (7.47% ± 2.02%) (Figure 4). Collectively, inhibition of LDH-A resulted in a decrease in the rate of proliferation of HuCCT-1 cells, while apoptosis rates increased. These results implicate the role of LDH-A in tumorigenesis.

### ROS levels associated with the LDH-A expression

**Figure 5:**
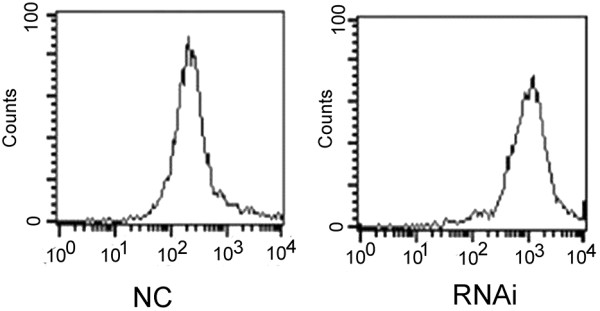
**Intracellular ROS production was detected with DCFDA fluorescence and monitored by flow cytometry at 72 hours post-transfection with shLDH-A (RNAi) or siControl (NC).** DCFDA, dichlorofluorescin diacetate; RNAi, RNA interference; ROS, reactive oxygen species; sh, short hairpin.

Silencing LDH-A has been reported to increase the OXPHOS capacity and to induce oxidative stress [6,14]. Therefore, we hypothesized that ROS, generated during respiration [16], might increase following a reduction in the expression of LDH-A in cholangiocarcinoma. ROS levels in the RNAi groups were three times higher than those in the NC group (Figure 5). These data suggest that LDH-A expression is linked to the ROS level of ICC and that inhibition of LDH-A increases ROS levels.

## Discussion and conclusions

The principal result of our experiments was the finding that the expression of LDH-A was higher in ICC. This result agrees with the observation of Wang *et al*. that the LDH-A gene is up-regulated in ICC cells compared to normal liver tissues [17]. An increase of LDH-A in tumor cells is linked with an aggressive phenotype in colorectal adenocarcinomas. In other studies of gastric carcinoma, the overexpression of LDH-5 was more prevalent in advanced tumors having positive vessel invasion, and patients with overexpression of LDH-5 showed far lower disease-free and overall survival rates compared with patients with low LDH-5 expression; therefore, LDH-A expression may be a useful prognostic factor for patients with gastric carcinoma [10,15]. Our study revealed a significant correlation between LDH-A and clinicopathologic characteristics (Table 1); poorly differentiated ICC cells tended to have higher expression of LDH-A. In conclusion, LDH-A is overexpressed in ICC and could, therefore, serve as an additional marker for malignancy.

Earlier studies have shown that attenuation of LDH-A expression causes impairment of tumor maintenance and leads to a reduction in tumor growth in xenograft models of renal cancer [6,18]. Here, we observed that knockdown of LDH-A in the ICC cell line HuCCT-1 is associated with the reduction of the proliferation rate and an increase in the apoptosis rate. Our research supports the hypothesis that the upregulation of LDH-A creates an adaptive advantage by which cancer cells evade apoptosis. The high expression of LDH-A in cancer cells causes cancer cells to use aerobic glycolysis rather than OXPHOS as their main means of glucose metabolism. This allows these cells to sustain higher proliferative rates and to resist some cell death signals, particularly those mediated by increased oxidative damage [14,19]. Therefore, cancer-specific isoforms of enzymes involved in the Warburg effect (such as LDH-A) may represent new drug targets for ICC treatment [20,21]. Individuals with a hereditary LDH-A deficiency show myoglobinuria only after intense anaerobic exercise (exertional myoglobinuria), and they do not show any symptoms under ordinary circumstances. So LDH-A may be, in principle, a specific, tolerated, and effective antitumor target for ICC.

These pro-apoptosis effects of the down-regulation of LDH-A in ICC could be attributed to a decrease in ROS. Reduction of LDH-A has been reported to promote oxidative phosphorylation [6], with the result of inducing apoptosis through the mitochondrial pathway [22]. Our finding that ROS levels increased remarkably in the RNAi group agreed with these reports. We infer that an increased sensitivity to oxidative stress may be a major mechanism for the pro-apoptotic effect of reduced LDH-A expression.

In general, the results of our experiments indicate that LDH-A is highly expressed in ICC and that inhibition of LDH-A causes remarkable apoptosis of the HuCCT-1 cell line. Therefore, LDH-A might be a specific therapeutic target for ICC. Furthermore, our results indicate that ROS levels are linked with the expression of LDH-A and may be the cause of the pro-apoptotic effect induced by the reduction of LDH-A expression in ICC.

## Abbreviations

7-ADD: 7-aminoactinomycin D; ICC: intrahepatic cholangiocarcinoma; LDH-A: lactate dehydrogenase-A; RNAi: RNA interference; ROS: reactive oxygen species; sh: short hairpin.

## Competing interests

The authors declare that they have no competing interests.

## Authors’ contributions

YY and ML carried out the molecular genetic studies, participated in the clinical studies and drafted the manuscript. ZF participated in the design of the study and performed the manuscript review. RL carried out the literature research and data acquisition. JC participated in the data analysis and statistical analysis. HF carried out the manuscript editing. All authors read and approved the final manuscript.
